# Breast Cancer Detection on Dual-View Sonography via Data-Centric Deep Learning

**DOI:** 10.1109/OJEMB.2024.3454958

**Published:** 2024-09-05

**Authors:** Ting-Ruen Wei, Michele Hell, Aren Vierra, Ran Pang, Young Kang, Mahesh Patel, Yuling Yan

**Affiliations:** Santa Clara University 7162 Santa Clara CA 95053 USA; Santa Clara Valley Medical Center 14454 San Jose CA 95128 USA

**Keywords:** Breast cancer classification, deep learning, radiologist comparison

## Abstract

*Goal:* This study aims to enhance AI-assisted breast cancer diagnosis through dual-view sonography using a data-centric approach. *Methods:* We customize a DenseNet-based model on our exclusive dual-view breast ultrasound dataset to enhance the model's ability to differentiate between malignant and benign masses. Various assembly strategies are designed to integrate the dual views into the model input, contrasting with the use of single views alone, with a goal to maximize performance. Subsequently, we compare the model against the radiologist and quantify the improvement in key performance metrics. We further assess how the radiologist's diagnostic accuracy is enhanced with the assistance of the model. *Results:* Our experiments consistently found that optimal outcomes were achieved by using a channel-wise stacking approach incorporating both views, with one duplicated as the third channel. This configuration resulted in remarkable model performance with an area underthe receiver operating characteristic curve (AUC) of 0.9754, specificity of 0.96, and sensitivity of 0.9263, outperforming the radiologist by 50% in specificity. With the model's guidance, the radiologist's performance improved across key metrics: accuracy by 17%, precision by 26%, and specificity by 29%. *Conclusions:* Our customized model, withan optimal configuration for dual-view image input, surpassed both radiologists and existing model results in the literature. Integrating the model as a standalone tool or assistive aid for radiologists can greatly enhance specificity, reduce false positives, thereby minimizing unnecessary biopsies and alleviating radiologists' workload.

## I. Introduction

Breast ultrasound (US) imaging, or sonography, is a non-invasive and safe imaging modality that complements mammography, the established gold standard for breast cancer screening. Diagnostic imaging typically employs orthogonal planes when evaluating anatomy and pathology [1], [2], [3], [4]. Unique to breast US imaging, the radial (RAD) and anti-radial (ARAD) axes are based on the underlying breast architecture and are the favored orientations for superior visualization and lesion comprehension, aiding in more effective diagnosis [1].

Unlike mammogram, US involves no ionizing radiation, making it particularly suitable for screening patients with dense breast, who are at higher risk [5], [6]. The device is also portable and cost-effective, although it may produce artifacts that can lead to false results, exceeding the error rate of mammography [5], [7], [8]. Deep learning (DL) has bridged this gap, matching the diagnostic capabilities of skilled radiologists [9], [10], [11]. However, the limited access to medical datasets due to patient privacy concerns is presenting challenges for the effective training of models. In this context, transfer learning has emerged as a solution by leveraging the capabilities of well-established convolutional neural network (CNN) models trained on large datasets such as ImageNet [12], a benchmark in computer vision, and RadImageNet [13], a radiology image repository in related medical domains. Researchers have increasingly embraced transfer learning by fine-tuning pre-trained CNN models such as VGG-[14] and GoogLeNet-based [15] architectures on a breast US dataset for breast cancer detection. [11], [16], [17], [18], [19], [20]. These efforts resulted in promising outcomes with their models yielding AUC scores ranging from 0.84 to 0.96. Notably, these studies primarily focused on single-view inputs, a trend seen in most studies using open-source datasets [21], [22]. On the other hand, Shen et al. [23], combining B-mode and Color Doppler images, achieved an AUC of 0.976 and aided radiologists to reduce false positive rates by 37%. Other studies handling 3D views (transverse, sagittal, and coronal), have reported AUC scores ranging from 0.82 to 0.951 [24], [25], [26], [27].

Notably, the publicly available breast US datasets [21], [22] often lack information regarding the image acquisition viewpoint or whether each image belongs to a distinct patient. Moreover, for some datasets, the groundtruth labels were obtained from Breast Imaging Reporting and Data System (BI-RADS) ratings, rather than being confirmed by biopsy. This labeling method introduces a potential source of uncertainty [28]. In our earlier work [29], [30], we tailored a CNN model first on an open-source dataset [21] and then evaluated it on a pilot dataset of dual-view breast US images. The model achieved an accuracy of 91.94%, sensitivity of 82.93%, and specificity of 96.39%. These results surpassed the performance of radiologists during blind evaluations of the same test set (71.7%, 73.6%, and 70.6% correspondingly). However, it is important to note that the dataset was handled as a single-view, unified dataset by consolidating the dual-views. This approach may potentially result in artificially inflated test results because the model learns at the image level rather than the patient level, introducing bias. We hypothesize that classifying based on both RAD and ARAD perspectives of a breast mass is more accurate than on a single perspective. Therefore, in this work we plan to conduct a comprehensive evaluation on the different assembling strategies of RAD and ARAD views and discover the optimal one for breast cancer classification.

Furthermore, several relevant review articles are available in the literature, with the latest one [31] conducting a comprehensive comparison between models and human readers regarding diagnostic accuracy on breast US images sourced from literature in 2018-2023. The review concluded that either standalone or as assistive tool, DL models consistently exhibited higher specificity when compared to human readers. The integration of DL into breast cancer diagnosis undoubtedly has the potential to transform clinical practice. Nevertheless, the lack of benchmark datasets and standardized research designs presents a barrier to progress in the field. Extensive research efforts are essential to fully unlock the positive impacts of this promising approach [31].

Our study prioritizes both model adaptation and data quality and relevance. The data-centric aspect emphasizes the importance of high-quality data and effective representation, which is now widely acknowledged as crucial [32], [33]. Leveraging an exclusive dual-view US image dataset provided to us by collaborators at Santa Clara Valley Medical Center (SCVMC), we pioneer an in-depth investigation into the pre-processing techniques and input representation strategies, with a focus on seamlessly integrating the dual views of breast images. Our objective is to enhance the specificity and sensitivity (or recall rate) in differentiating malignant from benign breast masses, by comparing to the radiologist's evaluation.

## II. Materials and Methods

This section outlines the methodology utilized in our research, including the dataset information, the preprocessing steps, the image cropping algorithm utilized, and the model adaptation.

### A. Dataset

**Fig. 1.**
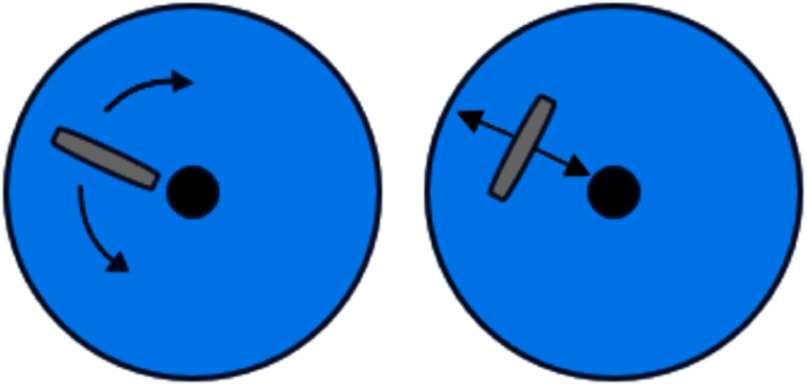
Dual-view image acquisition from RAD (left) and ARAD (right) axes.

This study has received institutional review board (IRB) approvals by both Santa Clara University's Sponsored Project Office (Protocol ID: 20-11-1526), and SCVMC following reviews by the Research & Human Subjects Review Committee (IRB Ref.#19-012). The dataset used is sourced from SCVMC. Representative images, in RAD and ARAD views, of 591 breast masses from 578 patients were selected by radiologists for inclusion. Fig. 1 illustrates the data acquisition method utilizing RAD and ARAD axes. Of the 591 masses, 398 were benign and 193 were malignant, which were confirmed through histopathologic diagnosis, followed by a biopsy process similar to the methodology described by Fujioka et al. [10]. Since each mass is represented by two images, there are 796 images of benign masses (398 RAD and 398 ARAD) and 386 images of malignant masses (193 RAD and 193 ARAD). We follow a 76.5%-13.5%-10% split to separate the dataset into training, validation, and test sets. To prevent bias, the dataset is divided at the mass level, ensuring that the RAD and ARAD views of the same mass do not appear in different sets.

### B. Image Preprocessing and Data Augmentation

**Fig. 2.**
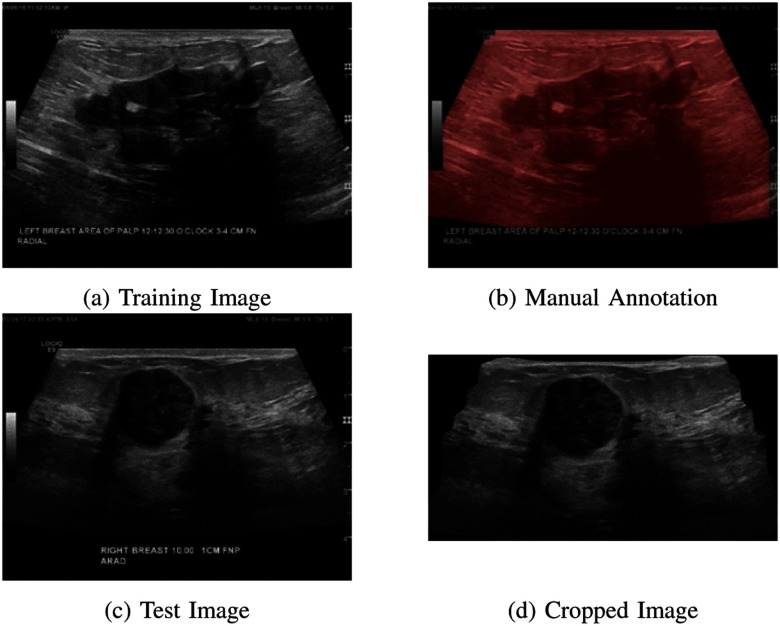
Image cropping process and results: (a) & (b) Original image and the annotated mask for training the Mask-RCNN model; (c) & (d) A test image and its mask generated by the model.

To maximize the model performance, we explore a series of image pre-processing techniques that encompass data augmentation and image cropping. Data augmentation, which is only applied to the training set, involves applying random horizontal and vertical flips to the original images, as well as making contrast adjustments during training. Furthermore, we perform image cropping to eliminate extraneous elements from the original images, such as text, scales, and rules. This is achieved through object detection using Mask-RCNN [34], a Mask Region-based CNN model, known for its proficiency in handling both object detection and instance segmentation tasks. In Fig. 2, an example image (Fig. 2(a)) is shown alongside its manually annotated mask (colored) overlaid on the original image (Fig. 2(b)). These mask annotations are utilized for training the Mask-RCNN model, allowing it to produce masks for new test images. Fig. 2(c) and 2(d) illustrate a test image and its cropped image, respectively, obtained by applying the mask generated by the model.

### C. Model Adaptation and Input Assembly Strategy

We tailored DenseNet169 [35] for transfer learning, which is known for its efficiency and effectiveness in image-related tasks such as classification. In particular, we initialize the model with pretrained weights to ensure smooth convergence of the model during training. Throughout our training process, we implement an early stopping mechanism to continuously track the validation loss and terminate the training if no improvement is observed over the past 10 epochs. Given the inherent class imbalance where benign masses outnumber malignant ones by more than two-fold, we assign a weight of “2” to the malignant class to effectively address this imbalance.

We outline the experiments we designed and conducted to explore various approaches of handling the dataset at the image-level (Experiment 1), as separate datasets of single view images (Experiments 2$\sim$3), or assembling dual views as model input (Experiments 4$\sim$8). Experiments 4 and 5 stitch the dual views horizontally or vertically into a single image, while Experiments 6$\sim$8 stack them along the channel, with various options as the third channel, as illustrated below.

**Fig. 3.**
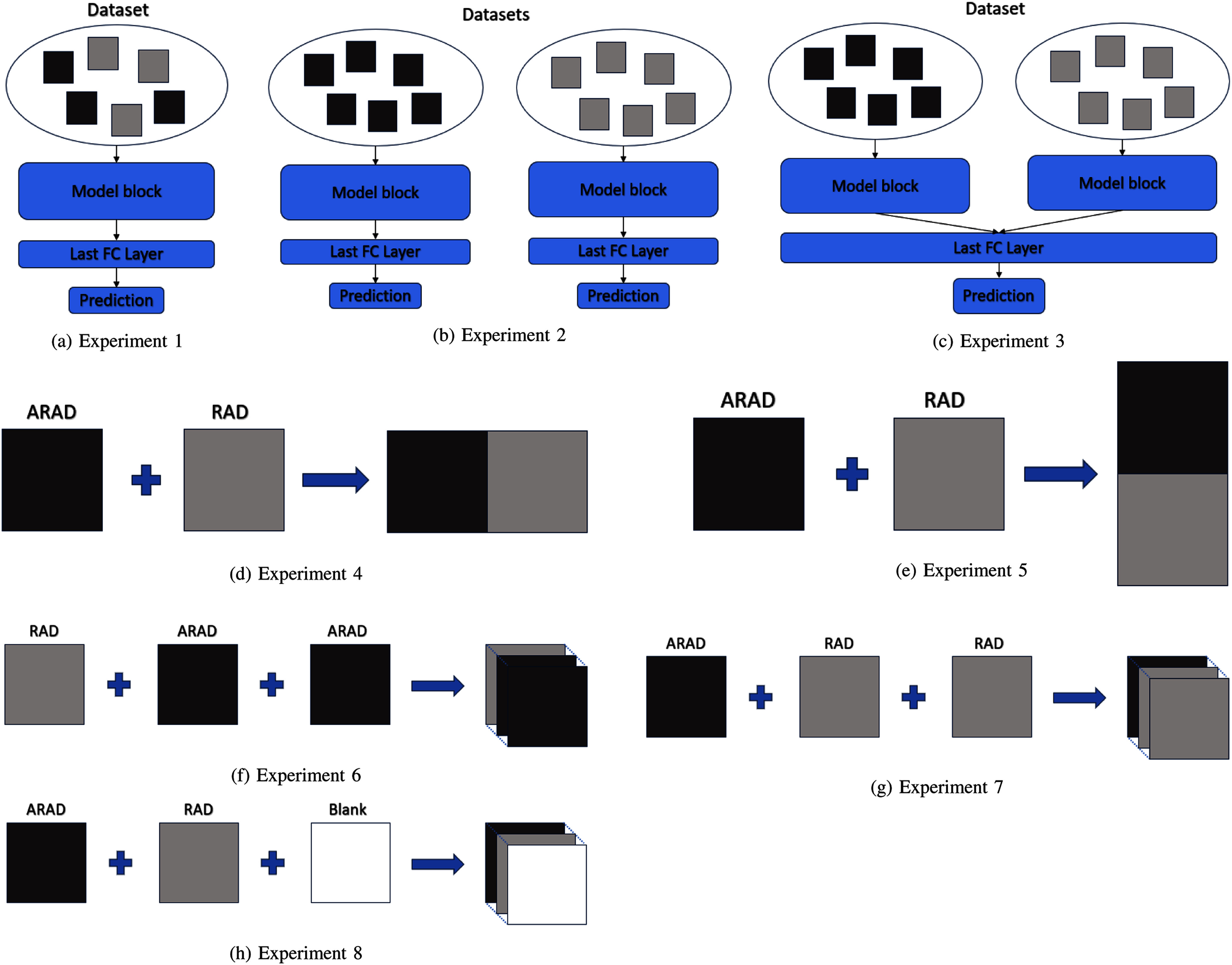
Rendering of input image configurations for all experiments showing how RAD and ARAD views are stacked or concatenated.

Experiment 6 & 7. Channel-wise stacking RAD and ARAD views while duplicating ARAD or RAD view respectively as the third channel. Experiment 8. Channel-wise stacking RAD and ARAD views while keeping one channel void, using a matrix of zeros. The design of all these experiments is illustrated in Fig. 3 for enhanced clarity and understanding.

## III. Results

In each experimental setup, we conducted binary classification to differentiate between benign and malignant masses. Subsequently, we present the mean and standard deviation for key performance metrics such as accuracy, precision, recall (sensitivity), specificity, and AUC. These findings are summarized in Tables I and II, using the original image inputs and the cropped image inputs respectively.

**TABLE I:** Experiment Results With Original Images (Mean $\pm$ Standard Deviation Over 5 Runs.)

Experiment	Accuracy	Precision	Recall	Specificity	AUC
1	0.8134 $\pm$ 0.01	0.7529 $\pm$ 0.01	0.6410 $\pm$ 0.02	0.8975 $\pm$ 0.01	0.8630 $\pm$ 0.01
2 (RAD)	0.7932 $\pm$ 0.05	0.7020 $\pm$ 0.09	0.6421 $\pm$ 0.10	0.8650 $\pm$ 0.06	0.8565 $\pm$ 0.03
2 (ARAD)	0.7763 $\pm$ 0.03	0.6712 $\pm$ 0.09	0.6526 $\pm$ 0.14	0.8350 $\pm$ 0.08	0.8388 $\pm$ 0.03
3	0.7254 $\pm$ 0.01	0.5878 $\pm$ 0.01	0.4947 $\pm$ 0.03	0.8350 $\pm$ 0.01	0.8167 $\pm$ 0.00
4	0.7187 $\pm$ 0.04	0.5778 $\pm$ 0.33	0.2526 $\pm$ 0.20	0.9400 $\pm$ 0.05	0.8045 $\pm$ 0.06
5	0.7153 $\pm$ 0.04	0.6729 $\pm$ 0.37	0.2105 $\pm$ 0.25	0.9550 $\pm$ 0.07	0.7722 $\pm$ 0.03
6	**0.9326** $\pm$ 0.03	**0.9126** $\pm$ 0.04	**0.8737** $\pm$ 0.06	**0.9600** $\pm$ 0.02	**0.9754** $\pm$ 0.01
7	**0.9153** $\pm$ 0.03	**0.8345** $\pm$ 0.06	**0.9263** $\pm$ 0.03	**0.9100** $\pm$ 0.04	**0.9538** $\pm$ 0.01
8	0.6881 $\pm$ 0.02	0.5245 $\pm$ 0.05	0.3684 $\pm$ 0.07	0.8400 $\pm$ 0.04	0.6629 $\pm$ 0.02

**TABLE II:** Experiment Results With Cropped Images (Mean $\pm$ Standard Deviation Over 5 Runs.)

Experiment	Accuracy	Precision	Recall	Specificity	AUC
1	0.7949 $\pm$ 0.00	0.7216 $\pm$ 0.01	0.6102 $\pm$ 0.02	0.8850 $\pm$ 0.01	0.8216 $\pm$ 0.00
2 (RAD)	0.7898 $\pm$ 0.01	0.7328 $\pm$ 0.01	0.5473 $\pm$ 0.04	0.9050 $\pm$ 0.01	0.8107 $\pm$ 0.01
2 (ARAD)	0.8136 $\pm$ 0.01	0.7996 $\pm$ 0.04	0.5684 $\pm$ 0.06	0.9300 $\pm$ 0.02	0.8609 $\pm$ 0.01
3	0.7831 $\pm$ 0.01	0.7083 $\pm$ 0.01	0.5579 $\pm$ 0.06	0.8900 $\pm$ 0.02	0.8580 $\pm$ 0.00
4	0.7017 $\pm$ 0.01	0.5517 $\pm$ 0.02	0.3895 $\pm$ 0.03	0.8800 $\pm$ 0.06	0.7301 $\pm$ 0.00
5	0.8136 $\pm$ 0.01	0.8231 $\pm$ 0.03	0.5368 $\pm$ 0.02	0.9550 $\pm$ 0.02	0.9487 $\pm$ 0.00
6	0.7966 $\pm$ 0.00	0.6522 $\pm$ 0.00	0.7895 $\pm$ 0.00	0.8000 $\pm$ 0.00	0.8521 $\pm$ 0.00
7	0.8000 $\pm$ 0.01	0.7820 $\pm$ 0.03	0.5263 $\pm$ 0.00	0.9300 $\pm$ 0.01	0.8087 $\pm$ 0.00
8	0.7661 $\pm$ 0.02	0.6678 $\pm$ 0.04	0.5473 $\pm$ 0.04	0.8700 $\pm$ 0.02	0.7970 $\pm$ 0.01

### A. Comparisons of the Model's Performance Across All Experiments

In our initial experiment, we tested a common method of combining both views into a single dataset, which can lead to a situation where a mass from the ARAD view is included in the training set while its corresponding RAD view is in the testing set. This bias, as highlighted by Han et al. [19], may falsely suggest improved performance. Our analysis showed a 2%$\sim$4% higher score in performance metrics for this approach compared to Experiment 2, where ARAD and RAD views were treated separately as individual inputs for each model. In Experiment 3, we utilized network fusion with a DenseNet backbone to process each view and concatenate their final layers to produce a single probability of malignancy for the breast mass. This approach, while similar to stacking, has limitations: the number of parameters is doubled but without an increase in data size, thus limiting the model's effectiveness. Due to subpar results and longer training times compared to alternative input configurations, this specific approach is not recommended.

Neither of the single-view approaches matched the performance achieved by incorporating dual views with a specific channel-wise stacking strategy in Experiments 6 or 7, highlighting the potential of dual views for more informative diagnosis. However, not all dual-view strategies consistently outperformed single-view approaches. For instance, arranging dual views side-by-side in Experiment 4 consistently yielded less favorable results, except for specificity, due to potential alterations in aspect ratio and shape, that are key features in distinguishing between benign and malignant masses [36]. In contrast, organizing dual views in a top-down manner (Experiment 5) produced notably improved results, especially with cropping. These outcomes surpassed single-view strategies, except for a slightly lower recall rate, noting that recall scores remained consistently low across all configurations. Interestingly, the same channel-wise stacking method but with the third channel void (Experiment 8) did not achieve the same level of performance as seen in Experiments 6 or 7.

To conclude, the channel-wise stacking of ARAD and RAD views with one of them duplicated as one channel (Experiments 6 and 7) yielded the best performance among all the strategies, with an accuracy of 0.9326 and 0.9152, a precision of 0.9126 and 0.8345, a recall (or sensitivity) of 0.8737 and 0.9263, a specificity of 0.96 and 0.91, and an AUC of 0.9754 and 0.9538, respectively. These scores significantly outperformed the results obtained from alternative image setups presented in this study that yielded an average accuracy of 0.7486, precision of 0.6413, recall of 0.4660, specificity of 0.8810, and AUC of 0.8020.

### B. Effects of Data Augmentation and Image Cropping

Our findings indicate that the implementation of the previously mentioned data augmentations has not yielded any noteworthy enhancements in the overall model performance. In contrast, when incorporating cropped images generated through Mask-RCNN for classification, we noticed improvements in certain image representations, although not in all cases. To illustrate, utilizing the cropped images resulted in a substantial enhancement of the classification in AUC for Experiment 5, with a notable increase from 0.7722 to 0.9487. Cropping also stabilized the training of side-by-side and top-down images, with the standard deviations decreasing from 0.2 or higher to 0.02 for precision and recall. As a side note, when employing a conventional method, such as maximum entropy for image cropping, we did not achieve any discernible enhancements across any of the experimental setups (results not displayed).

## IV. Discussion

### A. Comparing Model Performance With Radiologist Evaluations

**Fig. 4.**
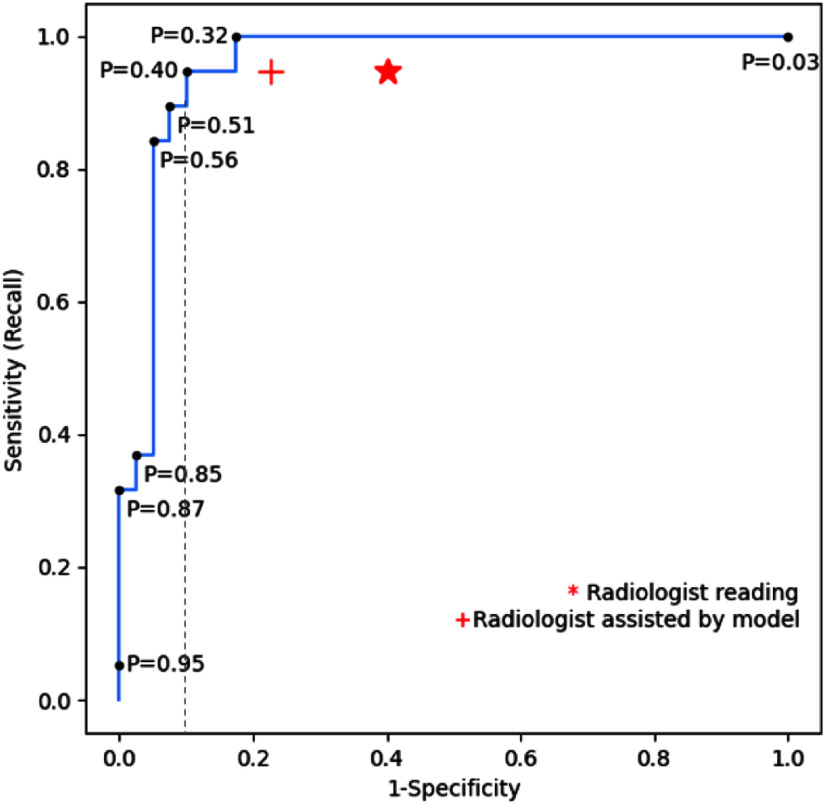
Performance comparison: ROC curve of model on dual-view input (in blue) vs. Radiologist reading (“*” in red) vs. Radiologist assisted by model (“+” in red). The vertical dotted line shows the corresponding false positive rate when the model achieved equal sensitivity as the radiologist.

While the model has achieved impressive performance, our next step is to assess its effectiveness by directly comparing key performance metrics with those of a human expert reader. We provided dual-view breast ultrasound images from the same test set used for model evaluation to a collaborating radiologist at SCVMC for blind evaluations. The test set comprises a total of 59 cases, including 19 malignant and 40 benign instances. Subsequently, we computed key metrics based on the radiologist's predictions compared to the corresponding biopsy-confirmed labels. These results yielded an accuracy of 0.7119, a precision of 0.5294, a recall of 0.9474, and a specificity of 0.6. Using a probability cutoff threshold (P) at 0.4, above which samples are classified as malignant, our model (Experiment 7) achieved equal recall and improved in other metrics: over 28% higher accuracy, 55% higher precision, and 50% higher specificity, as presented in Table III, when compared with the radiologist's performance. Fig. 4 displays the receiver operating characteristic (ROC) curve, which illustrates the multi-threshold performance of our model and visually helps in evaluating the trade-off between sensitivity and specificity of the model across the various threshold settings. It is clear that the radiologist's scores of sensitivity and specificity (marked with “*” in red) fall significantly below the curve, confirming the superior performance of the model. It is also evident from the Youden's index [37], which represents the maximum sum of (sensitivity + specificity -1) at an optimal cutoff threshold. Based on the ROC curve of our model, we identified the optimal cutoff threshold as 0.4. At this threshold, the model achieved the same sensitivity as the radiologist evaluation, and its corresponding specificity is 0.9 (see Fig. 4), which is significantly better than the specificity of the radiologist's evaluation at 0.6. Notably, when the cutoff threshold is at 0.32, both the sensitivity and specificity of the model are higher than the radiologist evaluation. It is evident that as we adjust the model's cutoff threshold to either maximize the sensitivity (P=0.32) or the sum of sensitivity and specificity (P=0.4), the model achieved better or equal performance in sensitivity while significantly improving the specificity, compared to the radiologist's reading. Furthermore, when assisted by the model predictions, the same radiologist enhanced his performance by achieving 17% higher accuracy, 26% higher precision, and 29% higher specificity (Table III), although these improvements still lag behind the standalone model performance, particularly in sensitivity and specificity (marked with “+” in red) that place below the ROC curve.

**TABLE III:** Performance Comparisons Across Four Metrics for Standalone Model (Experiment 7) Vs. Radiologist, and Radiologist Assisted by Model Vs. Alone.

	Model (P=0.4)	Radiologist	Improvement (Standalone Model)	Radiologist +Model	Improvement (Assistive Model)
Accuracy	0.9153	0.7119	+28%	0.8305	+17%
Precision	0.8182	0.5294	+55%	0.6667	+26%
Recall	0.9474	0.9474	0.00%	0.9474	0.00%
Specificity	0.9000	0.6000	+50%	0.7750	+29%

The Model Exceeded the Radiologist Under Many Metrics and Improved the Radiologist's Evaluation.

This direct comparison between the model and the human reader yields valuable insights into the importance of leveraging the DL model to support diagnostic decision-making. The integration of the model as either a standalone or an assistive tool for radiologists can lead to a significant decrease in false positives (cases incorrectly identified as malignant masses). This reduction offers a substantial benefit by avoiding unnecessary biopsies on benign lesions, which not only minimizes patient discomfort but also results in cost savings in healthcare.

### B. Case Studies

**Fig. 5.**
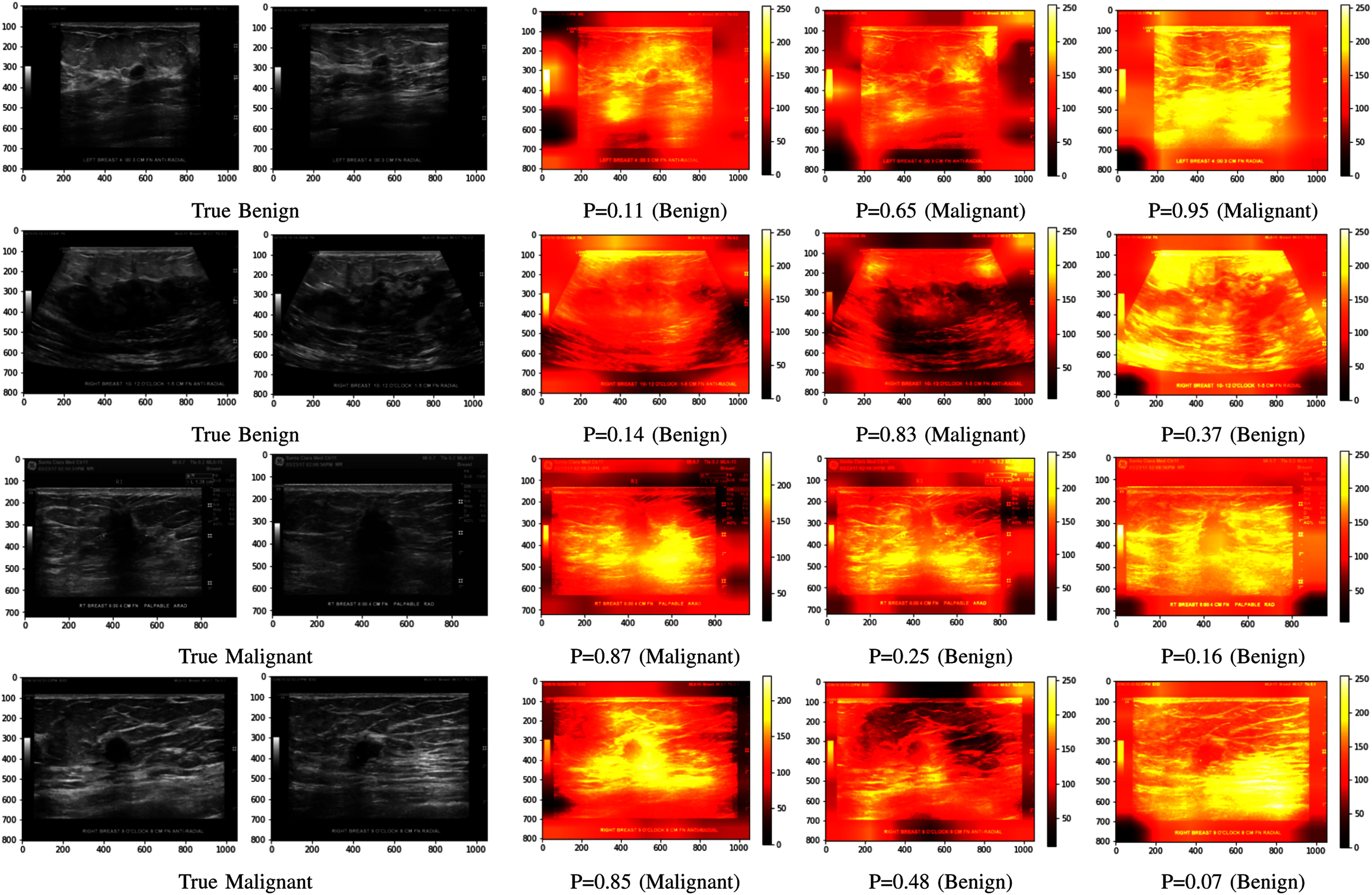
Comparisons between dual-view (middle column) and single-view approaches (right two columns) through class activation maps and predicted malignancy probabilities for four masses. Original ARAD and RAD images are shown in the left two columns.

In Fig. 5, we present two benign and two malignant instances from the test set, showcasing both RAD and ARAD views in the first and second columns, respectively. Our model, which employed an optimal channel-wise stacking strategy (Experiment 6) to incorporate the dual-views as input, accurately predicted all cases. The predicted malignancy probabilities were 0.11 and 0.14 for the benign cases, and 0.87 and 0.85 for the malignant cases. In contrast, these same cases were inaccurately predicted when the model was trained solely on either the ARAD- or RAD-view images.

To provide better insights into the model's decision-making process based on diverse input approaches, we use heat maps to elucidate its training behavior. Heat maps are powerful tools for visualizing the importance or intensity of features within an image. Specifically, the class activation map (CAM) [38] is invaluable for discerning which regions influence the model's classification output.

In Fig. 5, we illustrate four dual-view masses (first & second columns) where the model trained on the optimal dual-view stacking input made accurate predictions, while the single-view models failed. A threshold of 0.5 is used to interpret malignancy probabilities, categorizing values below this threshold as benign and those equal to or above as malignant. We included the CAM to highlight areas of significant activation during inference; the RAD model (fifth column) shows high activation over a large area, missing the critical region of the mass, while the ARAD model (fourth column) displays inconsistent patterns, focusing on incorrect areas. In contrast, the dual-view model (third column) emphasizes the mass region of interest, contributing to precise predictions. These findings highlight the performance enhancement from the strategic stacking of dual views.

## V. Conclusion

Being the pioneer in handling the dual-view breast US dataset, our study highlights the importance of integrating both model- and data-driven methodologies to enhance performance in diagnosing breast cancer using dual-view sonography. We meticulously designed and validated a DenseNet-based model for binary classification, leveraging our unique dataset comprising benign and malignant breast masses with biopsy-confirmed ground truth labels. While the model-centric approach is crucial, our investigation into optimizing model input representations of the dual views proved to be equally important. Through a series of rigorous experiments, we explored various assembly strategies to utilize the complementary information offered by the dual views, thereby facilitating more effective model learning. Our findings unveiled notable variations in key performance metrics across models with different input configurations.

The most favorable outcomes were achieved through a channel-wise stacking approach, where RAD and ARAD views were integrated into two channels, while duplicating one view in the third channel. This specific configuration yielded an accuracy of 0.933, specificity of 0.960, and sensitivity of 0.927. Moreover, the remarkable overall classification accuracy and discrimination of the model are highlighted by an impressive AUC value of 0.9754. This accomplishment surpasses or rivals the outcomes reported in existing literature (ranging from 0.82 to 0.976) that utilized both single-view and 3D-view datasets (transverse, sagittal, and coronal). Our model outperformed the radiologist's evaluation, with a 50% increase in specificity. With the model's assistance, the radiologist improved accuracy by 17%, precision by 26%, and specificity by 29%, achieving a 44% reduction in false positive rates. This reduction can help minimize unnecessary biopsies. Through innovative input strategies and state-of-the-art model architectures, our study sets a new standard in dual-view sonography-based breast cancer diagnosis, demonstrating the value of integrating RAD and ARAD views to improve diagnostic accuracy.

Conflict of Interest

We declare no conflict of interest.

Author Contributions

**T. W.:** Conceptualization, methodology development, data analysis, illustration, manuscript drafting, and revision. **M. H.:** Data analysis and illustration. **A. V.:** Data collection, illustration, and validation. **R. P.:** Data collection and preparation. **Y. K.:** Resource management and supervision. **M. P.:** Resource management and supervision. **Y. Y.:** Conceptualization, methodology development, supervision, resource management, and manuscript editing and revision.
